# Retracted: A Rare Pathological Entity of Multiple Calcified Hyperplastic Dental Follicles

**DOI:** 10.1155/2017/5149065

**Published:** 2017-03-30

**Authors:** Case Reports in Dentistry

At the request of the authors, the article titled “A Rare Pathological Entity of Multiple Calcified Hyperplastic Dental Follicles” [1] has been retracted. We were informed that the patient has withdrawn their consent after the article's publication. The contents of the article are no longer available online in order to protect the patient's confidentiality.

## References

[1] Gunawardane S., Kapugama K. (2016). A rare pathological entity of multiple calcified hyperplastic dental follicles. *Case Reports in Dentistry*.

